# Comparative morphological trade-offs between pre- and post-copulatory sexual selection in Giant hissing cockroaches (Tribe: Gromphadorhini)

**DOI:** 10.1038/srep36755

**Published:** 2016-11-07

**Authors:** Kate L. Durrant, Ian M. Skicko, Craig Sturrock, Sophie L. Mowles

**Affiliations:** 1 The University of Nottingham, School of Life Sciences, Nottingham, NG7 2RD, United Kingdom; 2 The University of Nottingham, School of Biosciences, Sutton Bonington Campus, LE12 5RD, United Kingdom

## Abstract

Sperm competition theory predicts that animals face a trade-off between investment in weaponry and investment in ejaculate composition. Within the Madagascan giant hissing cockroaches (Tribe Gromphadorhini) differences in morphology exist that may indicate differing strategies of male-male competition. We compared relative pronotal horn length using high-resolution X-ray CT scanning data, relative testes mass, and male-male agonistic behaviour between two species of hissing cockroaches, *Gromphadorhina oblongonota* and *Aeluropoda insignis*. The gross morphology and behaviour of these two species indicated that *G. oblongonota* is selected for pre-copulatory mate acquisition and that *A. insignis* is selected for post-copulatory sperm competition. We found evidence for a trade-off when investing in testes mass vs. horn length between the species. The large, aggressive *G. oblongonota* follows a strategy of greater investment in weapons at the expense of testes mass while the smaller, less-aggressive *A. insignis* invests in relatively greater testes mass and less in pronotal weapon length. We also found evidence of a trade-off within each species, where individuals invest more heavily in weapon length at the expense of testes mass. These findings support the predictions of pre- and post-copulatory competitive investment trade-offs for a relatively understudied Tribe of cockroaches.

Pre-copulatory sexual selection is one of the primary forces driving the evolution of extravagant phenotypic traits in males, including body-size and weaponry^1^,^2^. Sexual selection continues post-copulation if females mate multiply, known as sperm competition^3^. Males subject to both of these selective forces face a potential trade-off between energetically-demanding investment in pre-copulation access to females via weaponry, and competing post-copulation via investment in sperm numbers or quality^4^. Sperm competition models that incorporate the trade-off between expenditure on competitive pre- and post-copulatory male traits predict that as population-level sperm competition intensifies, more investment should be spent on testes and less on weaponry and mate searching^4^.

There is evidence for the predicted trade-off between pre- and post-copulation investment in males from organisms as diverse as Arctic charr (*Salevlinus alpinus*)^5^; domestic Fowl (*Gallus gallus domesticus*)^6^ and cetaceans^7^. Among invertebrates there is experimental evidence from dung beetles (*Onthophagus nigriventris*)^8^ and the cockroach *Nauphoeta cinerea*^9^.

The hissing cockroaches of Madagascar are large, heavily-armoured insects that engage in aggressive male-male contests over females^10^. They use complex acoustic and behavioural displays and have sexually dimorphic pronotal horns of varying pronouncement across species^10^,^11^. Although data is scarce, and there are no published accounts of the mating systems of wild Gromphadorhini, *Gromphadorhina portentosa* is thought to have a mating system characterised by dominance-based territoriality with female choice of high-ranking males^12^,^13^,^14^. Hissing cockroaches are thus predicted to face trade-offs in expenditure on weapons and ejaculates that will vary according to the ecological and social conditions that each species lives under. As with the exoskeleton, the development of testes and production of sperm is complete at adult emergence in *G. portentosa*^15^ meaning that investment allocations are fixed upon reaching sexual maturity.

In order to explore the nature of pre-and post-copulatory trade-offs we compared two species, the Wide-horned cockroach *G. oblongonota* (see Fig. 1a; video 1: https://figshare.com/articles/3D_rendered_animation_of_Gromphadorhina_oblongonota/3833892), and its monospecific sister genus, the Flat-horned cockroach *Aeluropoda insignis* (see Fig. 1b; video 2: https://figshare.com/articles/3D_rendered_animation_of_Aeluropoda_insignis/3833919)^16^, which vary in overall body size and the size and shape of pronotal horns as well as observed male-male aggression.

## Material and Methods

Thirty-three male *Aeluropoda insignis* and 46 male *Gromphadorhina oblongonota* were sourced from breeding colonies at the University of Nottingham. Cockroaches were housed individually as nymphs at 26 ± 1 °C, 12:12 h L:D cycle, fed *ad libitum* a diet of dry dog food and water and raised to maturity in isolation to ensure virginity. They were measured from the distal edge of the pronotal shield to the end of the last abdominal segment using callipers. Pairs of males size-matched by body-length were allowed to interact for up to 20 minutes in a 17 × 10 × 14.5 cm Perspex chamber under red light. Interactions were terminated early if dominance was quickly established to avoid damage to the animals. Interactions were filmed from above in HD video (Sony Handycam HDR-XR160, Japan) and reviewed at half-speed using VLC Media Player. Using the *G. portentosa* aggression ethogram^10^, aggressive behaviours were scored for each interaction in each species using JWatcher 1.0^17^. The behavioural events scored for each animal in the dyad were hissing, antennate opponent, butt opponent, flip opponent and abdomen thrashing^10^.

After data were gathered on male-male agonistic behaviour, cockroaches were euthanised by freezing. Euthanised cockroaches were stored at −80 °C and then defrosted at room temperature prior to CT-scanning. Post-scanning, cockroaches were dissected and combined testes wet weight was measured to an accuracy of 1 × 10^−5^g.

We utilised high-resolution X-ray computed tomography to gain precise morphological data. Prior to scanning, individual cockroaches were carefully wrapped in X-ray transparent polyethylene packing foam and placed in 50 mL centrifuge tube. Samples were scanned using a three section multi-scan in ‘fast mode’ using a GE v|tome|x M 240 kV| X-ray microCT system. The scan consisted of 2638 projection images acquired at 75 kV electron acceleration energy and 200 μA current with a detector timing of 250 ms. The spatial resolution of the scan was 30 μm. Images were reconstructed using Datos Rec v2.3.0 software (GE Inspection and Sensing Technologies, Germany) and VGStudio MAX 2.2 (Volume Graphics, Germany) was used to obtain total body volumes from each specimen.

We used ImageJ^18^ to obtain morphological measurements from the CT images from both species. Body length was measured dorsally from the distal leading edge of the pronotum to the tip of the last abdominal segment (see Supplementary Fig. 1a). The ‘freehand line tool’ was used to measure the length of the crenulated anterior edge of the pronotal shield in *A. insignis* to determine elaboration of this trait (see Supplementary Fig. 1b). For each species horn length was measured laterally from the lower distal corner of the pronotum to the apex of the horn (see Supplementary Fig. 1c,d).

All data were analysed using R version 3.1.0^19^. The R package ‘plotrix’ was used to produce standard errors of the mean, all means are reported ±1 standard error of the mean. To determine the shape of allometric relationships within and between species, testes mass and horn length were tested for relationships against body volume and body length respectively under three models to determine which was the best-fitting: (1) a simple linear model, (2) a polynomial model and (3) an exponential model; with species as a factor. The most parsimonious final model was selected using a backwards stepwise removal procedure, removing the higher-order interactions first and comparing against the last model calculated using ANOVA to determine whether the inclusion of the interaction term improved the model. The model with the highest Adjusted R^2^ when compared to the alternate model and with a significant p-value when tested against the null model for the trait in question was then selected as the best-fitting.

To correct for differing body size between the two species, each morphological trait was divided by a body size indicator (testes mass (g)/total body volume (mm^3^) and horn length (mm)/total body length (mm)) and used in a GLM with species as a factor. Thus the final model included relative testes mass as the dependent variable, relative horn length as the explanatory variable and species as a factor to determine if there was a trade-off between pre-copulatory selected traits, horns, and post-copulatory selected traits, testes, that differed in strength or direction between the two species.

## Results

Forty-two agonistic male-male encounters were recorded for each species, 19 in *Aeluropoda insignis* and 23 in *Gromphadorhina oblongonota*. The two cockroach species differed in the incidence of aggressive behaviours observed during male-male encounters. *G. oblongonota* was observed to engage in antennation, butting and flipping of opponents significantly more frequently than *A. insignis* (Table 1). *A. insignis* hissed more frequently during male-male encounters than *G. oblongonota* (Table 1). There was no significant difference between recorded incidences of abdomen thrashing. We did not score aggressive events per unit of time as there was no significant difference in the duration of male-male encounters between the two species (Wilcoxon rank sum test: W = 1036, n = 84, p = 0.139).

The two cockroach species differed significantly in average body length, horn length and combined testes mass. Mean body length of each species: *A. insignis* = 56.30 mm ± 1.30, *G. oblongonota* = 68.33 mm ± 1.14, Kruskal-Wallis Χ^2^_1_ = 33.82, p < 0.001. Mean horn length of each species: *A. insignis* = 4.04 mm ± 0.13, *G. oblongonota* = 7.22 mm ± 0.23, Kruskal-Wallis Χ^2^_1_ = 55.44, p < 0.001. Mean testes mass of each species: *A. insignis* = 0.04303 g ± 0.00205, *G. oblongonota* = 0.02826 g ± 0.00098, ANOVA, F_1,77_ = 50.16, p < 0.001.

**Figure 2:**
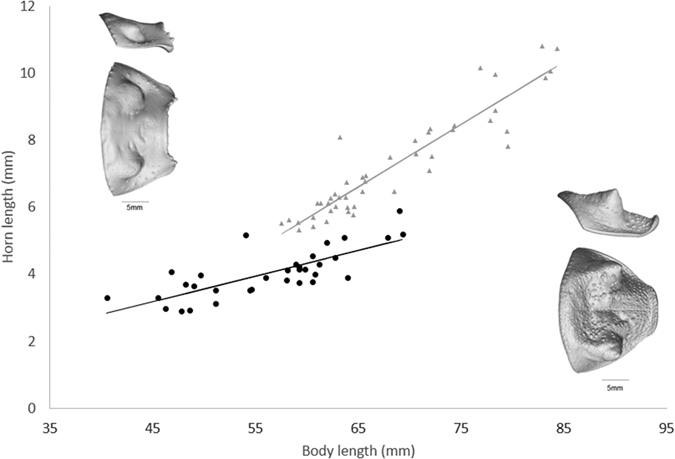
Relationship between total body length and pronotal horn length of *G. oblongonota*, (grey triangles, pronotal shield at bottom right in dorsal and lateral views) and *A. insignis*, (black circles, pronotal shield at top left in dorsal and lateral views). Trend lines are estimated using the original data and model parameters (Line equations for *A insignis*: y = 0.0765x −0.2665; *G. oblongonota*: y = 0.1865x −5.5199).

**Figure 3:**
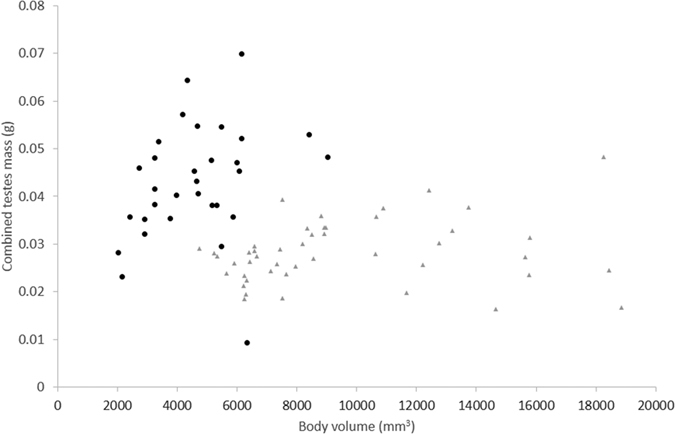
Relationship between total body volume and combined testes mass for *G. oblongonota* (grey triangles, n = 46) and *A. insignis* (black circles, n = 31).

Horn length in *A. insignis* was related to body length by a linear equation (Adjusted R^2^ = 0.5792, F_1,31_ = 45.05, p < 0.001), indicating horn size scales linearly with body size (Fig. 2). No models supported the fitting of any particular equation to the relationship between *A. insignis* testes mass and body volume, the null model was the best fitting (Adjusted R^2^ = 0.0455, F_1,29_ = 2.43, p = 0.13) indicating the lack of a strong allometric relationship between overall body size and testes mass (Fig. 3). Total body volume was not available for two specimens. Pronotum crenulation in *A. insignis* was related to body length by a linear equation (Adjusted R^2^ = 0.634, F_1,31_ = 56.49, p < 0.001), indicating crenulation or elaboration at the leading edge of the pronotum scales isometrically with body size.

Horn length in *G. oblongonota* was related to body length by a linear equation (Adjusted R^2^ = 0.8619 F_1,44_ = 281.8, p < 0.001), indicating horn size scales linearly with body size (Fig. 2). There was a significant difference in the relationship between horn length and body length between the two species, whereby *G. oblongonota* have relatively larger horns at smaller body sizes and the relationship rises at a steeper allometric slope than for *A insignis* (ANOVA Model 1: horn length ~ body length + species + body length:species vs. Model 2: horn length ~ body length + species, F = 43.571, p < 0.001). No models supported the fitting of any particular equation to the relationship between *G. oblongonota* testes mass and body volume, the best fitting model was the null (Adjusted R^2^ = 0.0077, F_1,44_ = 1.349, p = 0.252) indicating the lack of a strong allometric relationship between overall body size and testes mass (Fig. 3).

**Figure 4:**
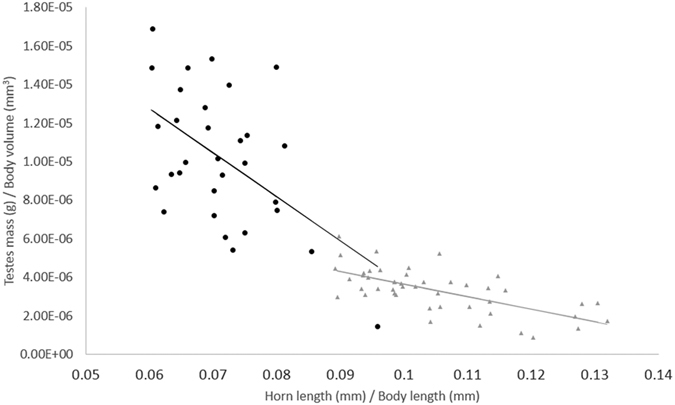
Relative testes size plotted against relative horn length for *A. insignis* (black circles, n = 31) and *G. oblongonota* (grey triangles, n = 46). Trend lines are estimated using the original data and model parameters (Line equations for *A insignis*: y = (−2.288e-04)x + 2.647e-05; *G. oblongonota*: y = (−6.48 e−05)x + 1.012 e−05).

There was a significant negative relationship between relative horn length and relative testes mass (Table 2). Across both species males that invest more in the size of their testes invest less in the length of their horns. Relative testes size differed significantly between the two species, *A. insignis* have relatively larger testes for their size than *G. oblongonota*, and also have greater absolute testes mass despite being the smaller species. There was a significant interaction between relative horn length, relative testes size and species (Table 2). In each species there was an apparent trade-off between testes size and horn length, with animals with relatively larger testes having relatively smaller horns, but the allometric slope of this relationship differs between the two species (Table 2, Fig. 4).

## Discussion

Morphometric analyses revealed that the smaller member of the Gromphadorhini tribe, *Aeluropoda insignis*, invested more in pronotal elaboration and horn length at larger body sizes. The linear relationship revealed isometric scaling of horn length with increasing body length. Similarly, isometry was observed in the elaboration of the leading edge of the pronotum. Testes mass was not allometrically related to body size, but absolute testes mass was greater in *A. insignis* than in *Gromphadorhina oblongonota*, and smaller male *A. insignis* invested more in their testes than smaller male *G. oblongonota*, indicating the relative importance of ejaculate-producing testes in competing post-copulation at all body sizes for this species. *A. insignis* males that cannot win in pre-copulation physical contests may have the opportunity to sneak copulations if females remain receptive and populations are aggregated.

*G. oblongonota* represents a competitive strategy with greater emphasis on weaponry. They are heavily armoured, and weapon development scales isometrically with body size while there is no relationship between testes mass and body volume. The lack of a clear relationship between body size and testes mass indicates that post-copulation competition is not as intense once dominance by male-male contest has been established. Therefore, males of this species do not invest proportionally in their testes as they attain larger body sizes, as would be predicted by isometric or positive allometric models. The small absolute size of the testes when compared to *A. insignis* also indicates that *G. oblongonota* is not likely to engage in intense post-copulatory sperm competition.

When comparing the two species it becomes apparent that while *A. insignis* is a significantly smaller animal with lesser investment in weaponry than *G. oblongonota*, they have significantly larger testes. Sperm competition theory^4^ suggests that this is a species that is operating under greater risk of post-copulatory competition and investing relatively more in their spermiogenesis organs than *G. oblongonota*. This may be related to variation in aggregation behaviour of naturally occurring populations of both species. Male Chorusing frogs (*Crinia georgiana*) that aggregate at higher densities invest less heavily in weaponry and more in counter-measures against sperm competition^20^. Natural aggregation patterns in our species are unknown, but in two other members of the Gromphadorhini tribe, *G. portentosa* and *Princisia vanwaerebeki*, males preferred not to aggregate, and female *P. vanwaerebeki* were also not gregarious, although female *G. portentosa* were^21^. This suggests the potential for differing aggregation patterns between species of this tribe.

The linear relationships between body size and weapon size in these cockroaches may appear surprising for traits suspected to be under sexual selection pressure. There is some expectation that if a morphological trait is under sexual selection then it should exhibit positive allometry^22^,^23^, however, positive allometry is not always the signal of a sexually selected trait. A meta-analysis by Bonduriansky^24^ demonstrated that isometry or negative allometry are the more commonly observed patterns within a range of sexually selected traits across taxa and suggested that well-known cases of positive allometry resulted from a sampling bias towards a minority of excessively exaggerated traits. Thus our observed isometric scaling is not that unusual when patterns from many taxa are considered. Allometric patterns can differ markedly across species, however. A recent meta-analysis^25^ estimated that weakly negative allometry may be the most common way that general traits scale with body size within species, but also found support for positive allometry in sexually selected traits, and interestingly, strong support for negative allometry in genital traits of insects and spiders^26^. Genital traits include the aedeagus or any other organ involved in sperm transfer, and are an important component of sperm competition that remains to be examined in the Gromphadorhini.

*A. insignis* were less aggressive during male-male interactions than *G. oblongonota*. More physical attacks such as butting and flipping were more frequent in the species with absolutely larger weapons, *G. oblongonota*, while agonistic signalling in the form of hissing was observed more frequently in *A. insignis*. Acoustic signalling is an important element of Gromphadorhini agonistic behaviour, it appears to convey information on body size to opponents^27^. We have shown that the use of acoustic signalling during fights differs between species of differing levels of aggression, with the less aggressive *A. insignis* hissing more frequently during male-male encounters, possibly as part of an assessment strategy that may enable small males to avoid physical violence^28^.

Differing levels of aggression and agonistic tactics may indicate different pre-copulation competitive strategies between the two species. Differing levels of aggression between species do not appear random, given the costly nature of agonistic behaviour. The species that displayed the highest level of aggression between three species of the Fence lizard genus *Sceloporus* was also the most dichromatic, and dichromatism was positively correlated with aggression^29^. This is another case of aggression correlating with greater elaboration of a sexually-selected trait across species, as we have demonstrated with aggression and horn length in *G. oblongonota*. Crickets, Orthoptera: Gryllidae, are often used as a model of male-male aggression, and a phylogenetic comparison of aggression across 13 species found that there were marked differences in inter-specific levels of aggression^30^. Bertram *et al*.^30^ suggested that high levels of male-male aggression in a cricket species was related to their tendency to use burrows to signal from, and that non-burrowing species were generally less aggressive. Aggression among males of the most aggressive Fence lizard species was attributed to low population densities that reduced the frequency of violent fights and high predation pressure selecting for residents that defended refuges^29^. These studies^29^,^30^ highlight the role of ecological data in aiding the interpretation of behavioural and morphological data, which are currently lacking for the Gromphadorhini.

## Conclusion

Males of the two giant hissing cockroach species appear to face the predicted trade-off between investing in pre- and post-copulatory competition, and each species addresses this trade-off in different ways. *A. insignis* follows a strategy of investing in testes while remaining relatively small-sized with reduced weaponry, and displaying lower levels of male-male aggression while *G. oblongonota* follows a strategy of large body-size, large weaponry and highly aggressive male-male encounters, but no specific investment strategy in testes and sperm production. These observations support sperm competition theoretical predictions but require field observations of natural mating systems in these relatively poorly-known organisms for confirmation. Thus, here we have identified a scenario of both within and between-species differentiation in mating strategies, whereby the two species may gain reproductive success through differential investment in spermiogenesis or weaponry.

## Additional Information

**How to cite this article**: Durrant, K. L. *et al*. Comparative morphological trade-offs between pre- and post-copulatory sexual selection in Giant hissing cockroaches (Tribe: Gromphadorhini). *Sci. Rep.* **6**, 36755; doi: 10.1038/srep36755 (2016).

**Publisher’s note**: Springer Nature remains neutral with regard to jurisdictional claims in published maps and institutional affiliations.

## Supplementary Material

Supplementary Information

Supplementary Information

## Figures and Tables

**Figure 1:**
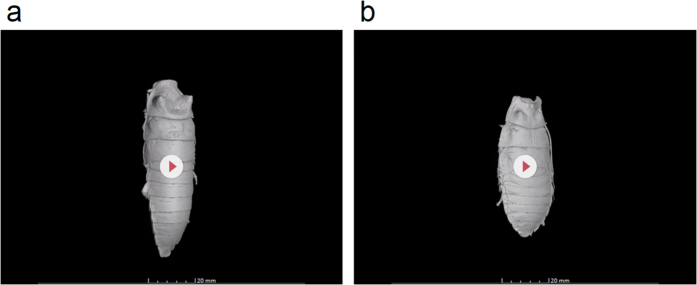
Screenshots from online videos demonstrating the morphological differences in overall body shape and pronotal shield weaponry between (**a**) *Gromphadorhina oblongonota* https://figshare.com/articles/3D_rendered_animation_of_Gromphadorhina_oblongonota/3833892 and (**b**) *Aeluropoda insignis* https://figshare.com/articles/3D_rendered_animation_of_Aeluropoda_insignis/3833919.

**Table 1:** Differences in the number of aggressive behaviours per male-male encounter between two species of hissing cockroach including Wilcoxon rank sum test statistics and associated p-values, with significant values in bold.

Behaviour	Species	n	Mean (S.E.M.)	Min.	Max.	W	p
Antennate	*A. insignis*	38	11.00 ± 3.84	0	104		
	*G. oblongonota*	46	61.37 ± 16.94	0	613	636	**0.0311**
Butt	*A. insignis*	38	0.79 ± 0.29	0	8		
	*G. oblongonota*	46	5.87 ± 1.45	0	40	560.5	**0.0021**
Flip	*A. insignis*	38	0.05 ± 0.05	0	2		
	*G. oblongonota*	46	0.80 ± 0.23	0	7	630	**0.0010**
Hiss	*A. insignis*	38	8.87 ± 4.17	0	130		
	*G. oblongonota*	46	1.04 ± 0.75	0	33	1052	**0.0262**
Abdomen thrashing	*A. insignis*	38	0.58 ± 0.50	0	19		
	*G. oblongonota*	46	3.76 ± 2.21	0	92	787	0.1476

**Table 2:** GLM results for relative testes size predicted by relative horn length with species as a factor.

Variable	Estimate (S.E.)	df	t value	p
Intercept	2.647e−05 (3.269e−06)	3, 73	8.097	<0.001
Relative horn length	−2.288e−04 (4.572e−05)	3, 73	−5.005	<0.001
Species	−1.635e−05 (4.247e−06)	3, 73	−3.848	<0.001
Relative horn length:Species	1.640e−04 (5.246e−05)	3, 73	3.126	0.0025
