# Supplementary figures and images for: Comparative morphological trade-offs between pre- and post-copulatory sexual selection in Giant hissing cockroaches (Tribe: Gromphadorhini)
Source: Sci Rep. 2016 Nov 7;6:36755. doi: 10.1038/srep36755 (PMC5098185; doi:10.1038/srep36755)

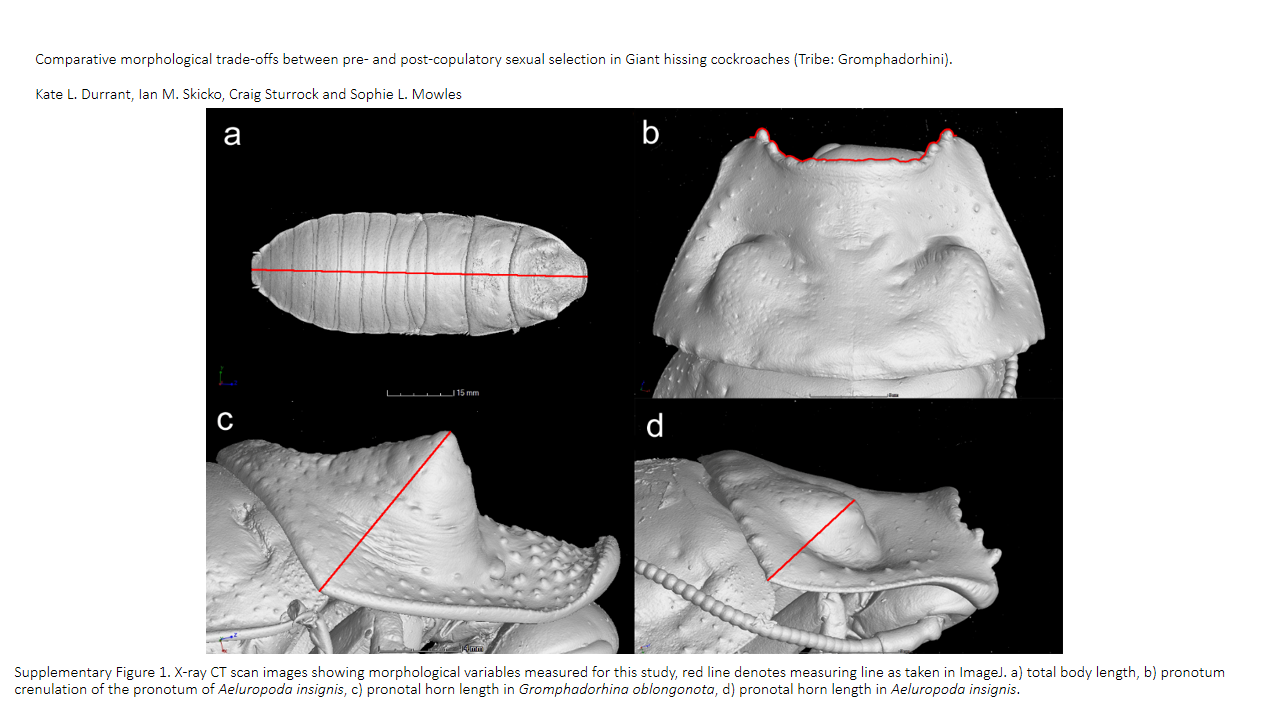

Supplement: Supplementary Information [file srep36755-s1.tiff]
